# Characterization of Carbide Precipitation during Tempering for Quenched Dievar Steel

**DOI:** 10.3390/ma15186448

**Published:** 2022-09-16

**Authors:** Yixin Xie, Xiaonong Cheng, Jiabo Wei, Rui Luo

**Affiliations:** 1 School of Materials Science and Engineering, Jiangsu University, Zhenjiang 212000, China; 2 Yangtze Delta Region Institute of Advanced Materials, Suzhou 215133, China

**Keywords:** Dievar steel, carbide precipitation, coarsening, hardness, toughness

## Abstract

Carbide precipitation and coarsening are investigated for quenched Dievar steel during tempering. Lath/lenticular martensite, retained austenite, lower bainite, auto-tempered, and larger spherical carbides are all observed in the as-quenched condition. The carbide precipitation sequence on tempering is ascertained to be: M_8_C_7_ + cementite → M_8_C_7_ + M_2_C + M_7_C_3_ → M_8_C_7_ + M_7_C_3_ + M_23_C_6_ → M_8_C_7_ + M_7_C_3_ + M_23_C_6_ + M_6_C; carbides become coarser on tempering, and the sizes for inter-lath carbides increase noticeably with increasing tempering temperatures due to the faster grain boundary diffusion, whereas the sizes for intra-lath carbides remain nearly constant. The rate of coarsening for carbides by tempering at 650 °C is much higher than those by tempering at 550 °C and 600 °C, due to the faster diffusion of alloying elements at higher temperatures.

## 1. Introduction

Hot-working die steel is exploited for molds and dies at working temperatures higher than the recrystallization temperature, to produce metal parts [1]. The failure for those molds and dies is predominantly due to thermal fatigue cracks, which are mainly because of inappropriate heat treatments or un-uniform distributions of primary carbides [2,3,4,5]. Composition optimizations and heat treatment modifications can help to prolong the service life of hot-working molds and dies.

Dievar steel is a new type of hot-working die steel that was designed based on the composition of H13 steel [6]. Compared to H13 steel, the remarkable differences in composition are in the decrease in Si content and the increase in Mo content. The reduction in Si can improve the steel’s ductility and toughness, with the presence of finer carbides [7]. A higher Mo content leads to higher hardenability, as well as the greater tempering resistance of steel [8]. The main failure modes are from cracking and plastic deformation caused by thermal fatigue [9,10]. The common heat treatment process for Dievar steel is spheroidization annealing, austenitizing, quenching, and tempering. The austenitizing temperature for Dievar should be in the temperature range of 1000–1070 °C [11,12]. Previous research showed that steels tested after oil-quenching at 1030 °C had a high hardness level and good wear resistance, but relatively lower impact toughness compared with samples quenched at other temperatures [13,14,15]. The undissolved carbides after oil quenching are all V-rich carbides with a face-centered cubic structure [16]. V-rich MC or M_8_C_7_-type carbides in Dievar remain at temperatures as high as 1050 °C, which normally originates from either the process of electroslag solidification or after remelting at 1030 °C [17]. Tempering in the temperature range from 400 to 700 °C is utilized to reduce or eliminate internal stresses for quenched hot-working dies. Rod-like cementite has been detected in an H13MOD (Fe-0.38C-5.13Cr-1.84Mo-0.49V) steel after twice-tempering at 600 °C for 2 h; with an increase in tempering temperature above 600 °C, the coarsening of cementite occurs, leading to a considerable decrease in hardness [16,18]. Stable alloy carbides with substantially higher enthalpies of formation are promoted with the strong carbide-forming elements (such as Mo, V, Cr, Ti) during tempering, contributing to the secondary hardening stage. In H13 steel, M_23_C_6_ + M_7_C_3_ (Cr-rich), MC (V-rich), and M_6_C (Mo-rich) carbides with large sizes have all been distinguished on tempering at relatively high tempering temperatures (above 600 °C) [16,19,20]. However, in the H13MOD steel, Mo-rich M_2_C carbides have been observed and remain at a very small size for tempering twice at 600 °C for 2 h, plus a further tempering at 620 °C for 2 h, contributing to the secondary hardening stage [16]. Finally, different precipitations of carbides on tempering at different temperatures directly result in the variation of mechanical properties, such as hardness and impact toughness [21,22,23,24,25,26,27,28].

Several efforts had been already made regarding heat treatments and microstructure evolutions for Dievar steel [6,14,29,30]; however, the detailed carbide precipitation and coarsening behaviors have not yet been systematically and quantitatively studied. In this research, various carbide phases, precipitate sizes, carbide chemical compositions, and the carbide crystal structures in quenched Dievar steel under different tempering conditions have been analyzed.

## 2. Materials and Experimental Procedures

After vacuum-smelting, electroslag, and ultra-fine treatment, Dievar steel was selected for the present study, and its chemical composition is shown in Table 1. Specimens with a size of 130 mm × 40 mm × 30 mm underwent isothermal spheroidizing annealing to produce uniform structures with fine and spherical particles before further heat treatment. The heat treatment processes are listed in detail in Table 2.

The microstructure and carbide composition were examined using a NovaNano 450 scanning electron microscope (SEM) (FEI Company, USA), Jeol 2100 transmission electron microscope (TEM) (JEOL, Tokyo), and Talos F200S scanning transmission electron microscopy (STEM) (Thermo Fisher Scientific, Waltham, MA, USA). SEM and bright-field (BF) images were used to observe the morphology of various carbides, while selected area diffraction patterns (SADP) were used to confirm the carbide types. High-angle annular dark-field STEM (HAADF-STEM) imaging with energy-dispersive X-ray spectroscopy (EDS) was utilized to ascertain the carbide compositions. SEM samples with a size of 10 mm × 10 mm × 5 mm were polished to a PO-S finish and etched in 4% natal (Macklin, Shang Hai, China) for 30 s. Thin foil specimens for TEM and STEM observations were cut from the center of the as-quenched and tempered samples, then they were ground with mesh abrasive papers (grit no. 400-800-1000-2000) to 70–80 μm. Then, a Gatan 695 ion-beam thinner (Gantan company, USA) was used to produce a thin area by reducing the thinning voltage from 7 keV to 3 keV. ImageJ analysis software (National Institutes of Health, Bethesda, USA) was used to obtain the length, width, and distributions of particles from SEM or TEM images. Approximately 1000 particles were measured for each assessed condition.

JMatPro software (Sente Software, UK) with a general steel database was employed to calculate carbide dissolution temperatures and equilibrium carbide volume fractions, as well as equilibrium carbide compositions.

## 3. Results

### 3.1. Microstructures of Quenched Dievar Steel

**Figure 1:**
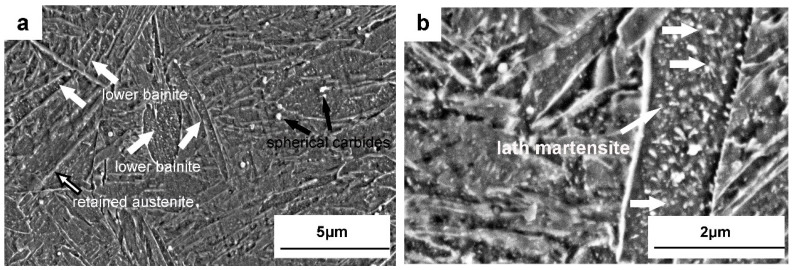
Microstructures of the Dievar steel after oil-quenching at 1030 °C, shown at different magnifications: (**a**) 15,000×, (**b**) 35,000×.

**Figure 2:**
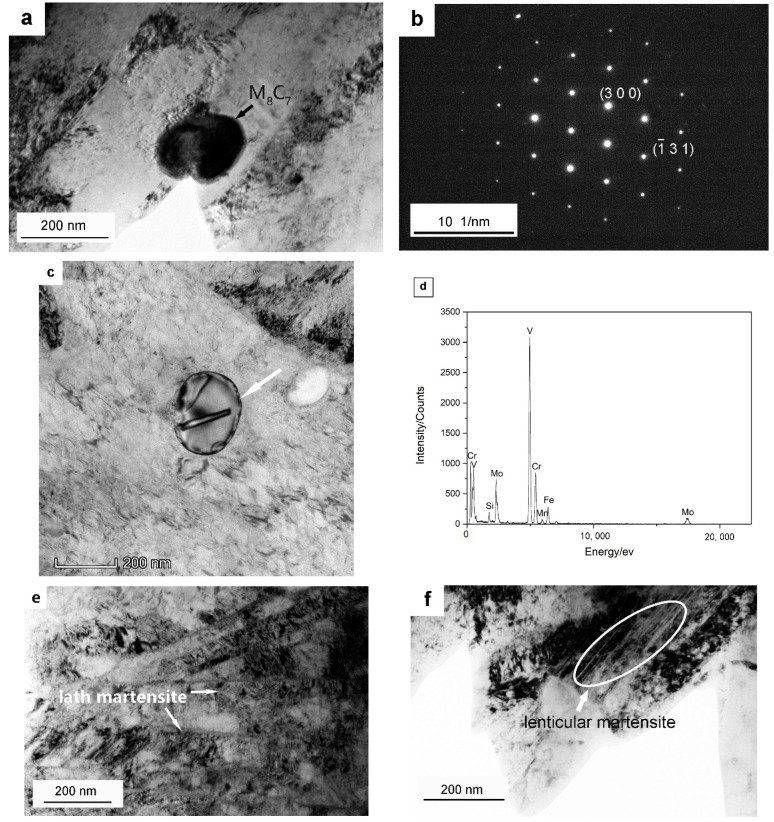
TEM images showing larger spherical carbides, lath martensite, and lenticular martensite after quenching at 1030 °C in Dievar steel: (**a**,**c**) bright field images of a typical larger spherical carbide; (**b**) SAD pattern for the particle in (**a**) consistent with the [3 −9 1] zone axis of M_8_C_7_; (**d**) EDS spectra for the particle in (**c**)**,** indicated by the white arrow; (**e**) lath martensite; (**f**) lenticular martensite.

Figure 1 shows the microstructures of the Dievar steel after oil quenching at 1030 °C with different magnifications, and Figure 2 shows the TEM images after quenching at 1030 °C in Dievar steel. Lath martensite, a small amount of lenticular martensite (4% volume fraction), retained austenite, a small amount of lower bainite (3.5% volume fraction), auto-tempered carbides, and larger spherical carbides have been all observed, as seen in Figure 1 and Figure 2. As shown in Figure 2a–d, these spherical carbides have been confirmed as V-rich M_8_C_7_ carbides, with a diameter ranging from 100 to 300 nm, as the dissolution temperature for V_8_C_7_ carbides has been predicted to be approximately 1050 °C, higher than the quenching temperature. Fine auto-tempered needle-shaped carbides with a length of ≤ 150 nm are located within martensite laths. The existence of these fine needle-shaped carbides demonstrates the occurrence of auto-tempering in this oil-quenched Dievar steel, which is most probably cementite [16]. Due to the high formation temperature of lath martensite, carbon still has a certain diffusion ability to segregate on dislocations and precipitated carbides after the formation of lath martensite during quenching [26,31].

### 3.2. Microstructure Evolutions during Tempering

**Figure 3:**
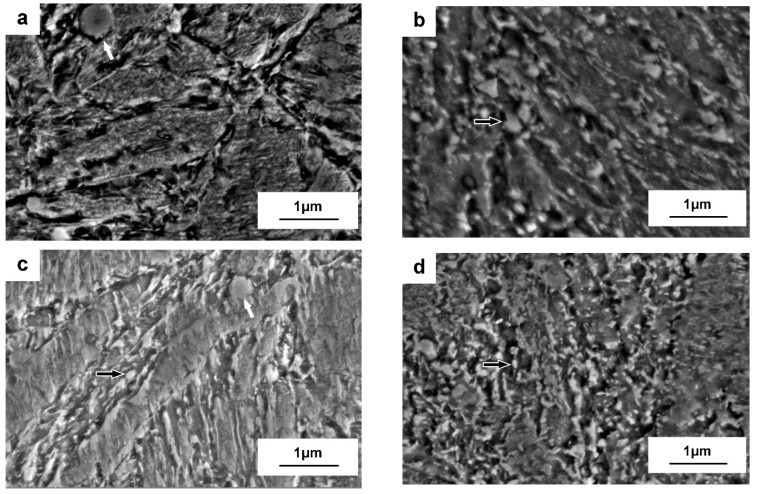

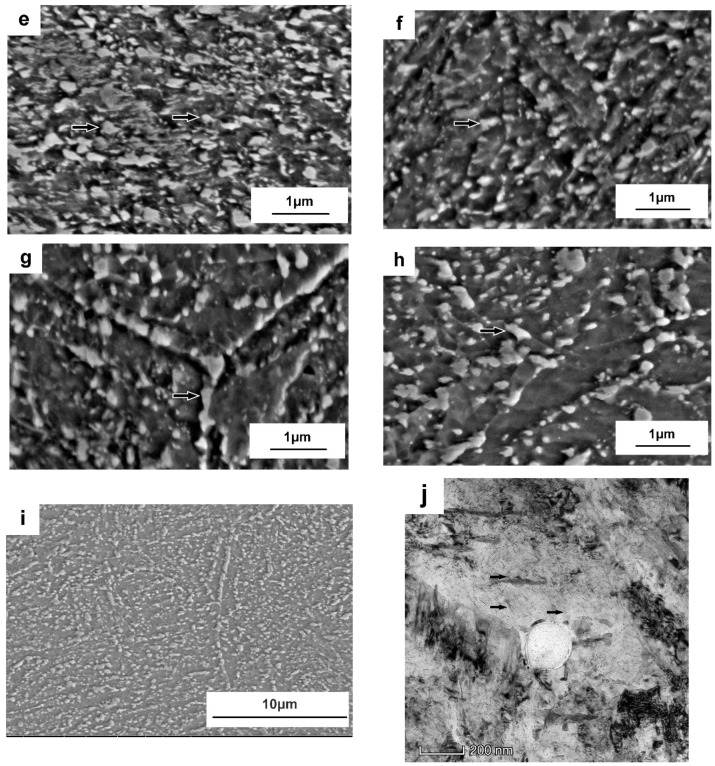
Morphology of Dievar steel after tempering at different temperatures for various time periods: (**a**) 550 °C for 4 h; (**b**) 550 °C for 24 h; (**c**) 600 °C for 4 h; (**d**) 600 °C for 16 h; (**e**) 600 °C for 24 h; (**f**) 650 °C for 4 h; (**g**) 650 °C for 16 h; (**h**,**i**) 650 °C for 24 h; (**j**) 600 °C for 4 h (TEM).

As shown in Figure 3a–h, martensite is then recovered, and the lath structure is regenerated and recrystallized during tempering at different temperatures with the extension of duration. As listed in Table 3, the coarsening of laths occurs more significantly at relatively higher temperatures, as the width of the laths grows more obviously on tempering at 650 °C compared to those with tempering at 550 °C and 600 °C. Recrystallization occurs in the tempered martensite at 650 °C for 24 h, since equiaxed ferrite grains have been observed (see Figure 3i).

Carbides with three morphologies (larger spherical particles, elliptical particles, and very tiny needle-shaped particles) are present in the matrix and along the boundaries after tempering at different temperatures for various times, as shown in Figure 3. Larger spherical carbides (indicated by the white arrows in Figure 3a,c) exist in the matrix and along the boundaries of tempering, which should be consistent with those samples in the as-quenched condition. Elliptical carbides (indicated by the black and white arrows in Figure 3b–h) have been observed after tempering for longer periods (such as 16 h and 24 h), due to the precipitation of larger alloy carbides and/or the coarsening and spheroidization of pre-existing carbides. Very tiny needle-shaped carbides with an average length of around 20 ± 5 nm (indicated by the black arrows in Figure 3j) precipitate within the martensite laths for tempering at 600 °C for 4 h.

**Figure 4:**
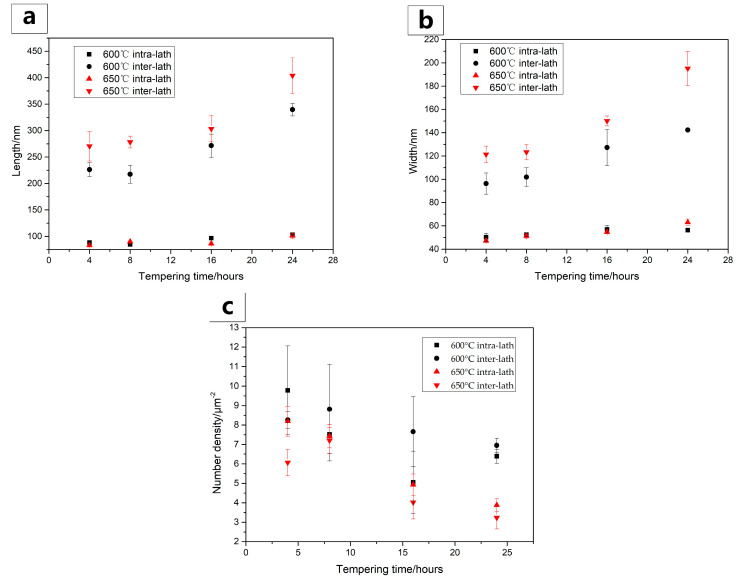
The average (**a**) length (**b**) width and (**c**) number density variations for carbides in Dievar steel in different tempering conditions (SEM observations).

Inter- and intra- lath carbides both coarsen and spheroidize during tempering; Figure 4 shows the inter- and intra-lath carbide size and number density variations for tempering at different temperatures. The sizes for inter-lath carbides are obviously larger than those for intra-lath carbides in Dievar steel, due to the faster solute lath/grain boundary diffusion [32]. It has also been shown that the size of inter-lath carbides increases noticeably with increasing tempering temperatures, e.g., from 600 °C and 650 °C (see Figure 4a). However, the size for intra-lath carbide keeps constant with increasing tempering temperatures (see Figure 4b), probably due to the newly precipitated alloy carbides replacing the previous larger particles. The carbide number density decreases with tempering time, due to the occurrence of carbide coarsening (see Figure 4c).

**Figure 5:**
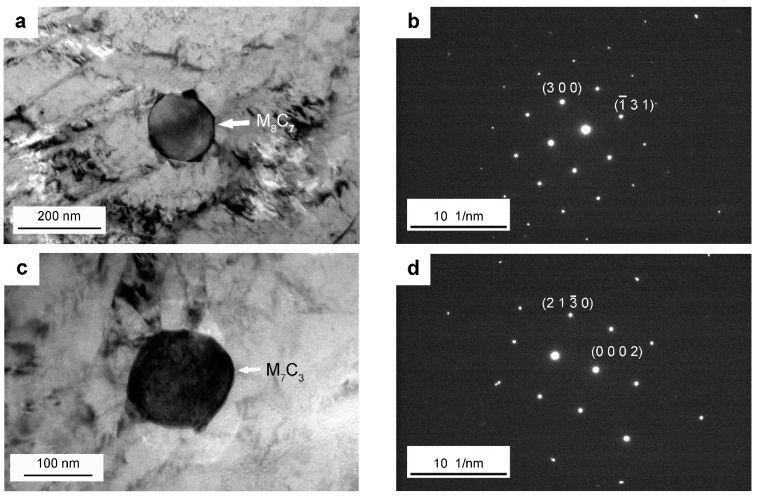

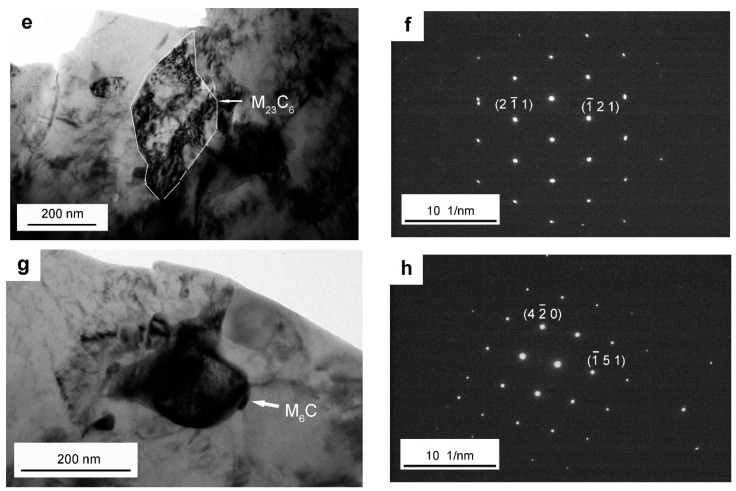
TEM images showing the presence of different alloy carbides tempered at 600 °C: (**a**) the morphology of M_8_C_7_ carbide (existing from 4 h to 24 h); (**b**) the corresponding SAD pattern for the particle in (**a**) consistent with the [0 −3 9] zone axis of M_8_C_7_; (**c**) the morphology of M_7_C_3_ carbide (existing from 4 h to 24 h); (**d**) the corresponding SAD pattern for the particle in (**c**) consistent with the [−4 5 −1 0] zone axis of M_7_C_3_; (**e**) the morphology of M_23_C_6_ carbide (existing from 16 h to 24 h); (**f**) the corresponding SAD pattern for the particle in (**e**) consistent with the [−1 −1 1] zone axis of M_23_C_6_; (**g**) the morphology of M_6_C carbide (solely existing for 24 h); (**h**) the corresponding SAD pattern for the particle in (**g**) consistent with the [−1 −2 9] zone axis of M_6_C.

**Figure 6:**
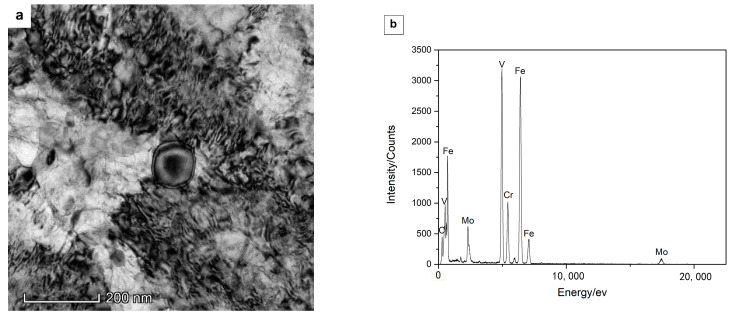

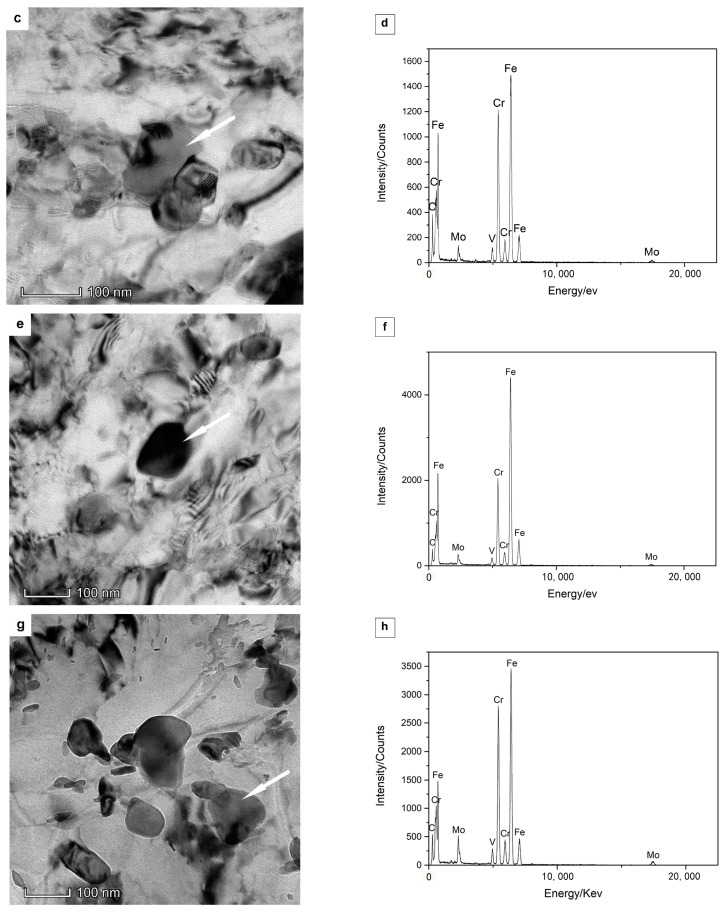
TEM images showing the different carbides present in the Dievar steel after tempering for 600 °C for different tempering times: (**a**) the spherical carbide (4 h); (**b**) EDX spectra for the spherical carbide in (**a**); (**c**) the smaller elliptical carbide (4 h); (**d**) EDX spectra for the elliptical (indicated by the white arrows) carbide in (**c**); (**e**) the elliptical carbide (16 h); (**f**) EDX spectra for the elliptical carbide (indicated by the white arrows) in (**e**); (**g**) different carbides with different shapes (24 h); (**h**) EDX spectra for the elliptical carbide (indicated by the white arrows) in (**g**).

**Figure 7:**
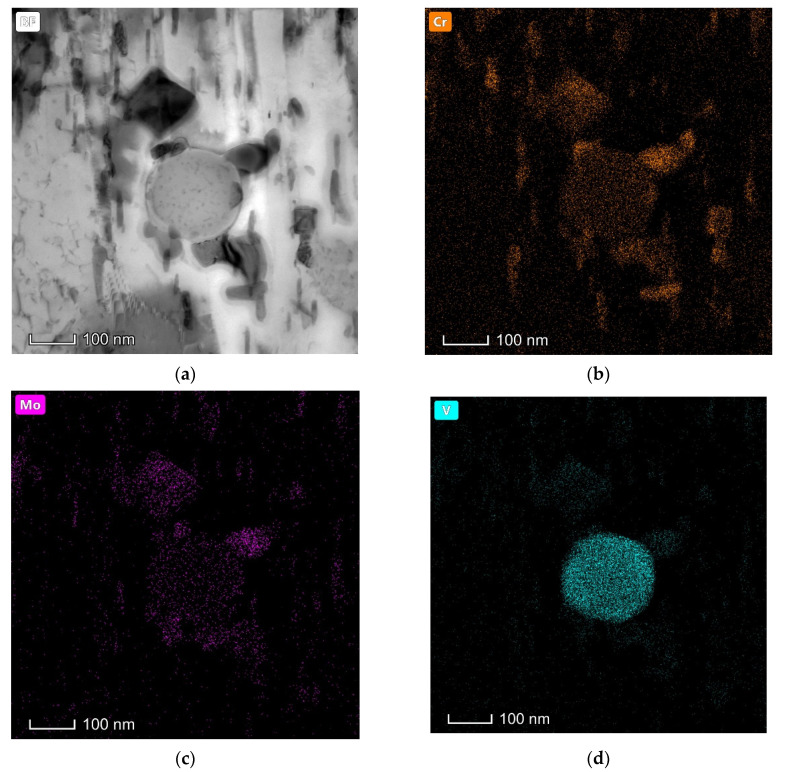
STEM EDS-mapping on different carbides precipitated upon tempering Dievar steel for 24 h: (**a**) The bright field image showing the existence of spherical, faceted, and rod-shaped carbides; (**b**–**d**) Elemental mapping.

Different types of alloy carbides have been distinguished after tempering at 600 °C (see Figure 5 and Figure 6). Spherical carbides remain within laths and along boundaries on quenching and tempering, which have been ascertained as V-rich M_8_C_7_ carbides (see Figure 5a,b and Figure 6a,b), being consistent with the literature reports on the carbide precipitation behavior for H13 steel [20]. Slightly smaller elliptical particles with a length of 100–200 nm start to be detected after tempering at 600 ºC for 4 h and have been confirmed as Cr-rich M_7_C_3_ carbides, as shown in Figure 5c,d and Figure 6c,d. Cr-rich M_7_C_3_ carbides precipitate not only along boundaries but also at the interface of pre-existing particles (such as M_8_C_7_ or cementite) in the matrix (Figure 6c). This is due to the diffusion of substitutional elements at this comparatively high temperature, such as Cr and Mo, contributing to the transformation from metastable cementite to alloy carbides. Very tiny needle-shaped particles have also been observed within laths after tempering at 600 °C for 4 h (Figure 3j) and are expected to be Mo-rich M_2_C carbides, according to the reports of the precipitation of Mo_2_C carbides in an H13MOD steel on tempering at 600 °C for 2 h twice, plus a further tempering at 620 °C for 2 h, which finally contributes to the resistance to hardness decrease and secondary hardening [16]. After tempering at 600 °C for 16 h and 24 h, most of the intra-lath carbides coarsen, as can be seen in Figure 5e and Figure 6g, and tend to have a larger elliptical shape with a diameter of around 150 ± 20 nm. These carbides have been determined as Cr-rich M_23_C_6_ carbides, which are also calculated as one of the equilibrium carbides in Dievar steel with a volume fraction of 6.4% on tempering at 600 °C. As listed in Table 4, the enrichment of Cr in carbides promotes the transformation from M_7_C_3_ to M_23_C_6_ for a relatively longer tempering process. In addition, Mo-rich M_6_C carbides have been found after a prolonged tempering time of 24 h in Dievar steel, except for M_8_C_7_, M_7_C_3_, and M_23_C_6_ (Figure 5g,h). As shown in Figure 7, faceted and elliptical Cr- and Mo-rich particles precipitate around the pre-existing spherical V-rich M_8_C_7_ particles, demonstrating that Cr- and Mo-rich carbides tend to nucleate at the interface of pre-existing alloy carbides. The precipitation sequence during tempering for the quenched steel can then be identified as follows: M_8_C_7_ + cementite (quenching) → M_8_C_7_ + M_2_C + M_7_C_3_ → M_8_C_7_ + M_7_C_3_ + M_23_C_6_ → M_8_C_7_ + M_7_C_3_ + M_23_C_6_ + M_6_C, which is generally consistent with the thermodynamic calculated equilibrium carbides, M_6_C, M_23_C_6_, and M_8_C_7_.

## 4. Discussion

The carbides in the as-received condition aligned in one direction, indicating the occurrence of elemental segregation. The segregation is weakened sharply after spheroidizing annealing as the carbides distribute more uniformly, this being consistent with a similar observation for H13 steel [33]. Cr- and Mo-rich carbides are detected after spheroidizing annealing in Dievar steel, where more Cr enriches alloy carbides compared to Mo, illustrating that Cr-rich M_23_C_7_ and M_7_C_3_ are expected to be the predominating carbides, compared with Mo-rich carbides. Mo-rich carbides probably form at the interface of M_7_C_3_ carbides during the transition of Cr-rich M_7_C_3_ to stable M_23_C_6_ in H13 hot-work tool steel [34].

Lath martensite is mainly formed after quenching in the Dievar steel because of the comparatively low carbon content (0.39 wt %). It has been found that the quenching speed of oil should be higher than 10 °C/s to avoid entering into the bainitic transition phase zone, based on the calculation of the CCT curve. Lower levels of bainite can still be still observed in Figure 1a, probably due to the large sample size (130 × 40 × 30 mm) and the lower cooling rate. The cooling rate is estimated to be lower than 8 °C/s [34] for the temperature region of 300~350 °C with oil-quenching. A small amount of retained austenite has also been observed. In addition, lenticular martensite has been observed in the quenched Dievar steel as well (Figure 2d). Steels with a carbon content of 0.3~1.0 wt % usually contain two kinds of martensite at the same time (lath martensite and lenticular martensite) and the carbon content of this steel at a temperature of 1030 °C in the matrix is predicted to be 0.368 wt %. The addition of the alloying elements Cr, Mo, and Mn also increases the tendency to form lenticular martensite [35]. Two types of carbides are observed in Dievar steel after quenching: spherical V-rich M_8_C_7_ carbides and needle-shaped cementite, formed via auto-tempering during oil-cooling. The theoretically calculated formation temperature of V-rich M_8_C_7_ is as high as 1050 °C, and the V_8_C_7_ that is detected experimentally is probably formed during electroslag solidification. Auto-tempered cementite particles have been observed within martensite laths (Figure 1b). Due to the high formation temperature of lath martensite and its slow cooling rate, carbon still has a certain diffusion ability during quenching and segregates into dislocations and precipitated particles [26,31].

The precipitation sequence for this steel after 1030 °C oil-quenching and 600 °C tempering can be identified as M_8_C_7_ + cementite (oil-quenching) → M_8_C_7_ + M_2_C + M_7_C_3_ → M_8_C_7_ + M_7_C_3_ + M_23_C_6_ → M_8_C_7_ + M_7_C_3_ + M_23_C_6_ + M_6_C. The V-rich M_8_C_7_ carbides observed in tempered microstructures are expected to originate from electroslag solidification [17] or quenching; for that reason, the dissolution temperature of M_8_C_7_ is higher than the austenitization temperature for Dievar steel, based on the thermodynamic calculations. In addition, the coarsening of M_8_C_7_ occurs relatively sluggishly with tempering, due to the slow diffusivity of V (e.g., D_V-α_(600 °C) = 1.4744 × 10^−14^ m^2^/s) [36]. Very tiny needle-shaped carbides with an average length of around 20 ± 5 nm that are present within the laths are expected to be Mo-rich M_2_C carbides with tempering at 600 °C for 4 h, which keeps them coherent with the matrix, leading to resistance to hardness decrease. M_2_C carbides are metastable carbides and are easily decomposed to stable M_6_C carbides with prolonged tempering time (e.g., 24 h). Cr-rich M_7_C_3_ carbides have appeared with tempering at 600 °C for 4~24 h, and it is expected that M_7_C_3_ carbides have been formed via the transformation of cementite, due to the enrichment of Cr in cementite and the nucleation on the cementite/ferrite interface [21]. In addition, it has been predicted via the JMatPro thermodynamic calculation that Cr-rich M_23_C_6_ is one of the equilibrium carbides at 600 °C in Dievar steel. The faceted Cr-rich M_23_C_6_ carbides have been observed on tempering for 16 h and 24 h, where YCr in Table 4 has achieved an equilibrium value of 1.53 with prolonged tempering time. M_23_C_6_ carbides are distributed along the boundaries and around the pre-existing V-rich M_8_C_7_ carbides in the matrix due to the segregation of alloying elements. In high Cr-Mo-containing steels [21], the precipitation sequences for Cr-rich carbides can be established as cementite → M_7_C_3_ → M_23_C_6_ or cementite → M_23_C_6_. Therefore, it is expected that stable M_23_C_6_ carbides form via the in situ transformation of M_7_C_3_ and/or cementite from a longer tempering time and/or higher tempering temperatures in this steel. Besides, Mo-rich M_6_C has been found after tempering at 600 ºC for 24 h, which is believed to be transformed from M_2_C. The coarsening of carbides occurs and tempered martensite recrystallizes on tempering from 4 h to 24 h, where the rate of coarsening for carbides after tempering at 650 °C is much higher than those after tempering at 550 °C and 600 °C, due to the faster diffusion of alloying elements at higher temperatures (i.e., D_Mo-__α_(600 °C) = 1.2694 × 10^–14^ m^2^/s, D_Mo-__α_(650 °C) = 1.427 × 10^−13^ m^2^/s) [36].

## 5. Conclusions

Carbide precipitation and coarsening behaviors during tempering in quenched Dievar steel have been quantified; the effect of the alloying elements, Cr, Mo, and V on the carbide transformation for tempering has been considered. Based on the carbide characterization, the main conclusions are:

(1) Microstructures after oil-quenching consist of lath martensite, lenticular martensite, retained austenite, a small amount of lower bainite, auto-tempered needle-shaped carbides, and larger spherical V-rich M_8_C_7_ carbides.

(2) The carbide precipitation sequence in quenched Dievar steel during tempering is identified as follows: M_8_C_7_ + cementite → M_8_C_7_ + M_2_C + M_7_C_3_ → M_8_C_7_ + M_7_C_3_ + M_23_C_6_ → M_8_C_7_ + M_7_C_3_ + M_23_C_6_ + M_6_C. Mo-rich unstable M_2_C carbides can easily decompose to equilibrium M_6_C. M_23_C_6_ carbides are expected to transform from M_7_C_3_ carbides and/or cementite. Cr, Mo-rich carbides distribute around the pre-existing V-rich carbides in the matrix and along grain/subgrain boundaries, which is probably due to the segregation of alloying elements.

(3) Carbides coarsen on tempering, whereas the sizes for inter-lath carbides are obviously larger than those for intra-lath carbides, due to the faster solute lath/grain boundary diffusion. The sizes for inter-lath carbides increase noticeably with increasing tempering temperatures, e.g., from 600 °C to 650 °C, whereas the sizes for intra-lath carbides keep nearly constant.

## Figures and Tables

**Table 1:** The chemical composition of Dievar steel (wt %).

Material	C	Mn	P	S	V	Mo	Cr	Si	Ni
Dievar	0.39	0.42	0.007	0.001	0.7	2.37	4.84	0.22	0.10

**Table 2:** The heat treatment processes.

Heat Treatment	Detailed Process
Annealing	860 °C for 2 h → furnace cooled to 740 °C and hold for 4 h → furnace cooled to 500 °C → air cooled to room temperature
Quenching	900 °C for 1 h → 1030 °C for 30 min → oil-quenched.
Tempering	550 °C for 4 h, 8 h, 16 h, 24 h, respectively → air cooled to room temperature 600 °C for 4 h, 8 h, 16 h, 24 h, respectively → air cooled to room temperature 650 °C for 4 h, 8 h, 16 h, 24 h, respectively → air cooled to room temperature

**Table 3:** Lath size variation in different tempering conditions.

Tempering Conditions	Lath Size/μm
550 °C + 4 h	0.8–1.9
550 °C + 24 h	1.1–2.0
600 °C + 4 h	0.9–2.5
650 °C + 4 h	1.6–3.4

**Table 4:** The M to Fe ratio Y_M_ (M = Cr, Mo, and V) for the different carbides in Figure 7 from the EDS measurements on tempering at 600 °C.

	Y_Cr_	Y_Mo_	Y_V_
The spherical carbide in (a)	0.16 ± 0.01	0.25 ± 0.01	0.86 ± 0.02
The smaller elliptical carbide in (c)	0.69 ± 0.02	0.16 ± 0.01	0.06 ± 0.01
The elliptical carbide in (e)	0.48 ± 0.01	0.06 ± 0.01	0.03 ± 0.01
The larger elliptical carbide in (g)	1.19 ± 0.04	0.31 ± 0.01	0.10 ± 0.01
